# Possible protective effect of green tea intake on risk of adult leukaemia

**DOI:** 10.1038/sj.bjc.6604140

**Published:** 2007-12-18

**Authors:** M Zhang, X Zhao, X Zhang, C D'Arcy J Holman

**Affiliations:** 1 The School of Population Health, The University of Western Australia, Perth, WA, Australia; 2 Department of Hematology, Second Affiliated Hospital, College of Medicine, Zhejiang University, Hangzhou 310009, PR China

**Keywords:** adult leukaemia, case–control study, green tea, risk factor

## Abstract

In a case–control study of 107 adults with leukaemia and 110 orthopaedic controls in China, a reduced risk was found with longer duration, higher quantity, and frequency of green tea intake.

Tea is one of the most frequently consumed beverages in the world (Graham, 1992). Different processing of tea leaves yields green, black, or Oolong tea. If tea leaves are immediately heated, then the enzymes that oxidise catechins are inactivated and the result is green tea; if crushed and allowed to undergo enzyme-mediated oxidation, the process results in black tea with its characteristic colour and taste. Oolong tea results from partially oxidised tea leaves (Graham, 1992).

There is some evidence of a protective effect of green tea consumption on certain cancers (Zhang *et al*, 2002; 2004; 2007; Cabrera *et al*, 2006), but no epidemiological data are available on the effect of green tea on adult leukaemia. We therefore conducted a case–control study of the question in southeast China.

## MATERIALS AND METHODS

A hospital-based case–control study was conducted in Hangzhou, southeast China. Eligible cases were defined as Chinese, over 15 years old and resident in Zhejiang Province with leukaemia, histopathologically confirmed according to the haematological standards (Zhang, 1998). They were identified by daily searching of medical records in haematology wards at two teaching hospitals of Zhejiang University in 2005–2006; all medical records and laboratory reports were also reviewed to ensure complete ascertainment of cases. A total of 107 patients aged 16–81 years were recruited, most (83.2%) were recent and were interviewed within 12 months of diagnosis. During the same period of data collection, 110 orthopaedic patients without leukaemia or other malignancy were randomly selected as controls to match the cases by age using a daily update of the list of cases. The project received approval from the hospital authorities and from the Human Research Ethics Committee of The University of Western Australia.

Subjects were briefed regarding the aims of the study and confidentiality issues. After obtaining their consent, a face-to-face interview was conducted in the hospitals using a structured, validated questionnaire on (a) demography and lifestyle; (b) tea consumption; (c) hazard exposure and family history of malignancy. Most cases were recent patients interviewed within 3–6 months of diagnosis. Details of leukaemia subtype and stage were retrieved from hospital records.

Tea consumption was measured using a questionnaire adapted from our previous studies, its reproducibility evaluated in a test–retest study where the intraclass correlation coefficient was 0.83 for tea (Zhang *et al*, 2005). Information was sought on preparation method, type of tea drank, duration of each type, frequency of cups (350–400 ml), frequency of new batches of tea brewed, quantity of dried tealeaf consumed per year (in *liang*, equivalent to 50 g), and date of cessation. Frequency of cups was categorised as never or seldom, once a month, 2–3 times a month, once a week, 2–3, 4–6 times a week, once a day, 2–3, and ⩾4 times a day.

### Statistical analysis

The data were coded and analysed using the SPSS package. As none of the participants reported drinking black or Oolong tea only and few (3.8%) drank both green and black tea, all tea drinkers were classified as green tea drinkers. All comparisons used a *t-*test for continuous variables and *χ*^2^-test for categorical variables. Odds ratios (ORs) of adult leukaemia and associated 95% confidence intervals (CIs) for green tea consumption as an explanatory variable were estimated using unconditional logistic regression. Multivariate models included potential confounders identified using univariate analysis and risk factors reported in other studies (Miligi *et al*, 1999; Ross *et al*, 2002; Kasim *et al*, 2005).

## RESULTS

Of leukaemia subtypes, 72 (67.3%) were acute myeloid leukaemia (AML), 22 (20.6%) acute lymphocytic leukaemia (ALL), 10 (9.3%) chronic myeloid leukaemia (CML), and 3 (2.8%) chronic lymphocytic leukaemia (CLL). Selected characteristics are shown in Table 1. Cases were more educated, more exposed to chloromycetin, benzene, and organophosphorus, but fewer were smokers, especially among the men. Notably, only 42.1% of cases were tea drinkers compared with 55.4% of controls. Among the 106 tea drinkers, 96.2% drank green tea only. No other differences were found in the characteristics covered in Table 1.

Selected characteristics of green tea drinkers and nontea drinkers are presented in Table 2. Compared with nontea drinkers, green tea drinkers tended to be older and more often male (72 *vs* 54%). There was no significant difference in residential area, education, or smoking.

Compared with nontea drinkers or infrequent tea drinkers, the adjusted ORs and associated 95% CIs were 0.39 (0.17–0.91), 0.20 (0.06–0.60), 0.40 (0.19–0.82), and 0.42 (0.20–0.86) in those who consumed green tea at ⩾1001 g of dried tea leaves per annum, for >20 years, at ⩾1 cup a day, and at ⩾1 new batches a day, respectively (Table 3). A significant dose–response relationship between green tea consumption and adult leukaemia risk was observed across all measures; the reduced risk was seen for ALL and CML/CLL combined, but not for AML.

## DISCUSSION

This case–control study found that higher intake of green tea was associated with a reduced risk of adult leukaemia and with significant dose–response relationships, the first such evidence, although a US hospital-based case–control study recently reported a negative association between black tea and AML in women (Li *et al*, 2006). Our findings are consistent with experimental evidence that green tea polyphenols inhibited proteasome and induced apoptosis in leukaemic cells (Smith *et al*, 2002).

The few identified risk factors in adult leukaemia account for only a small proportion of cases and include ionising radiation, exposure to benzene, pesticides, immunosuppression, chemotherapy, and smoking (Miligi *et al*, 1999; Ross *et al*, 2002; Kasim *et al*, 2005). We found no significant association of smoking with leukaemia risk, the proportion of smokers being higher in the controls. No association was found between AML and green tea intake.

The case–control design may have introduced bias and the study size was relatively small, although recruitment was carefully conducted. The response rate was high, and the matched controls were randomly selected from the same hospitals. Most patients in China are self-referred, and the recruitment procedure ensured that cases and controls came from the same populations. Selection bias among controls from convenience or clinician contact was also minimised.

Details were obtained on tea consumption as well as on lifestyle factors and exposures using a validated instrument, designed for residents in southeast China. Our research interest in any type of tea consumption was revealed at the time of the interview to explain why the questions on tea were so detailed. We consider the likelihood of any information bias to have been low, and it is relevant that there had been no mention in the popular media of any link between tea and leukaemia.

We found a higher proportion of nontea drinkers than in previous studies and that tea drinking was associated with older age. Although tea consumption can be reported with reasonable accuracy, misclassification may still occur. However, when nondifferential, such errors are likely to bias results towards the null and could not account for the significant inverse associations. Exposure of cases to risk factors may change due to disease status, but most were newly diagnosed within 3–6 months.

In conclusion, this study suggests that a higher intake of green tea is associated with a reduced risk of adult leukemia, but the finding needs to be checked in larger studies.

## Figures and Tables

**Table 1:** Selected characteristics of cases and controls

	**Cases (*n*=107)**	**Controls (*n*=110)**	**P-value^a^**
*Age at interview (years)*	42.9±16.4	44.5±16.9	0.46
15–<30	25 (23.3)	24 (21.8)	
30–<45	34 (31.8)	34 (30.9)	
45–<60	28 (26.2)	27 (24.5)	
⩾60	20 (18.7)	25 (22.7)	
			
*Gender*			0.77
Male	66 (61.7)	70 (63.6)	
Female	41 (38.3)	40 (36.4)	
			
*Locality of resident*			0.37
Metropolitan	29 (27.1)	24 (21.8)	
Suburb or rural area	78 (72.9)	86 (78.2)	
			
*Education*			0.02
No formal education	12 (11.2)	6 (5.4)	
Primary	24 (22.4)	29 (26.4)	
Secondary	49 (45.8)	66 (60.0)	
Tertiary	22 (20.6)	9 (8.2)	
			
*Tobacco smoking*			
Male smokers	29 (43.9)	45 (64.3)	0.02
Female smokers	0 (0)	3 (7.5)	0.07
			
*Overall tea consumption*	45 (42.1)	61 (55.4)	<0.05
Green tea only	45 (42.1)	57 (51.8)	
Black tea only	0 (0)	0 (0)	
Oolong tea only	0 (0)	0 (0)	
Green and black tea	0 (0)	4 (3.6)	
Medication use of chloromycetin	6 (5.6)	0 (0)	0.01
Occupational benzene exposure	16 (15.0)	1 (0.9)	<0.001
Occupational organophosphorus exposure	5 (4.7)	0 (0)	0.02

Values expressed as mean±s.d. or number (%).

a Two-sided *t-*test for continuous variables and *χ*^2^-test for categorical variables.

**Table 2:** Selected characteristics of green tea drinkers and nontea drinkers

	**Green tea drinkers (*n*=106)**	**Nontea drinkers (*n*=111)**	**P-value^a^**
Age at interview (years)	48.1±14.9	39.5±17.2	<0.001
			
*Gender*			<0.01
Male	76 (71.7)	60 (54.1)	
Female	30 (28.3)	51 (45.9)	
			
*Locality of resident*			0.19
Metropolitan	30 (28.3)	23 (20.7)	
Suburb or rural area	76 (71.7)	88 (79.3)	
			
*Education*			0.51
No formal education	9 (8.5)	9 (8.1)	
Primary	28 (26.4)	25 (22.5)	
Secondary	51 (48.1)	64 (57.7)	
Tertiary	18 (17.0)	13 (11.7)	
			
*Tobacco smoking*			0.40
Male smokers	43 (56.6)	31 (51.7)	
Female smokers	1 (3.3)	2 (3.9)	

Values expressed as mean±s.d. or number (%).

a Two-sided *t-*test for continuous variables and *χ*^2^-test for categorical variables.

**Table 3:** Associations between green tea consumption and the risk of adult leukaemia

	**No. of cases**	**No. of controls**	**Adjusted OR (95% CI)^a^**
*Tea drinking*			
No	62	49	1.0 (referent)
Yes	45	61	0.51 (0.27–0.96)
*P*_trend_^b^			0.04
			
*Duration (years)*			
0	62	49	1.0 (referent)
⩽10	20	21	0.68 (0.30–1.53)
>10 to ⩽20	11	17	0.66 (0.26–1.72)
>20	14	23	0.20 (0.06–0.60)
*P*_trend_^b^			<0.01
			
*Number of cups*			
Nondrinkers or ⩽1 time a week	67	50	1.0 (referent)
2–6 times a week	10	13	0.40 (0.14–1.14)
⩾1 time a day	30	47	0.40 (0.19–0.82)
*P*_trend_^b^			<0.01
			
*Number of new batches*			
Nondrinkers or ⩽1 time a week	67	50	1.0 (referent)
2–6 times a week	11	14	0.35 (0.12–1.01)
⩾1 time a day	29	46	0.42 (0.20–0.86)
*P*_trend_^b^			0.01
			
*Dried tea leaves (g year*^−*1*^)			
0	62	49	1.0 (referent)
⩽500	14	7	1.98 (0.67–5.81)
501–1000	12	16	0.20 (0.07–0.62)
⩾1001	19	38	0.39 (0.17–0.91)
*P*_trend_^b^			<0.01

CI=confidence interval; OR=odds ratio.

a Estimates from separate unconditional logistic regression models included terms for age (years; continuous), gender (male, female), residence (metropolitan, suburb or rural), education (none, primary, secondary, tertiary), smoking (never/ever), medication use of chloromycetin (no/yes), occupational exposure to benzene (no/yes), and organophosphorus (no/yes).

b Two-sided test for trend across quantitative or ordinal quantitative variables.
